# Integrated Metagenomic and Physiochemical Analyses to Evaluate the Potential Role of Microbes in the Sand Filter of a Drinking Water Treatment System

**DOI:** 10.1371/journal.pone.0061011

**Published:** 2013-04-11

**Authors:** Yaohui Bai, Ruiping Liu, Jinsong Liang, Jiuhui Qu

**Affiliations:** State Key Laboratory of Environmental Aquatic Chemistry, Research Center for Eco-Environmental Sciences, Chinese Academy of Sciences, Beijing, People’s Republic of China; U. S. Salinity Lab, United States of America

## Abstract

While sand filters are widely used to treat drinking water, the role of sand filter associated microorganisms in water purification has not been extensively studied. In the current investigation, we integrated molecular (based on metagenomic) and physicochemical analyses to elucidate microbial community composition and function in a common sand filter used to treat groundwater for potable consumption. The results revealed that the biofilm developed rapidly within 2 days (reaching ∼10^11^ prokaryotes per gram) in the sand filter along with abiotic and biotic particulates accumulated in the interstitial spaces. Bacteria (up to 90%) dominated the biofilm microbial community, with Alphaproteobacteria being the most common class. Thaumarchaeota was the sole phylum of Archaea, which might be involved in ammonia oxidation. Function annotation of metagenomic datasets revealed a number of aromatic degradation pathway genes, such as aromatic oxygenase and dehydrogenase genes, in the biofilm, suggesting a significant role for microbes in the breakdown of aromatic compounds in groundwater. Simultaneous nitrification and denitrification pathways were confirmed as the primary routes of nitrogen removal. Dissolved heavy metals in groundwater, e.g. Mn^2+^ and As^3+^, might be biologically oxidized to insoluble or easily adsorbed compounds and deposited in the sand filter. Our study demonstrated that the role of the microbial community in the sand filter treatment system are critical to effective water purification in drinking water.

## Introduction

Sand filter technology is commonly used throughout the world to treat drinking water. The effectiveness of this treatment mechanism is usually associated with physicochemical processes such as filtration and disinfection, while the role of microbial communities in removal of hazardous substance and water quality improvement is often ignored or underestimated. As water percolates through the sand, abiotic and biotic particulates accumulate in the interstitial spaces. As biomass builds, a biofilm eventually forms on the surface of sand grains [1]. The established biofilm can play a critical role in the transformation or degradation of a variety of harmful elements and compounds that occur in drinking water. Detailed analyses of biofilm, therefore, would provide information contributing to the understanding of the complex and fundamental interactions between the biological and physico-chemical processes operating within the sand filter [2].

Historically, assessments of microbial communities relied on culture-dependent techniques to infer the potential biological process in drinking water treatment systems. Recent research developments have overcome many of the technical limitations of the past, allowing for the use of certain molecular methods that target 16S rRNA to detect microbial community composition, thus providing a better description of biological mechanisms [3], [4], [5]. Despite a substantial expansion of our knowledge of microbial taxonomy and diversity in water treatment systems, the fundamental biological mechanisms driving water purification remain poorly defined. Metagenomics is a recent and rapidly developing field that attempts to analyze the genetic content of whole communities [6], thus providing a simultaneous assessment of the phylogenetic composition and metabolic pathways of microbial populations [7]. Recent advances in next-generation sequencing (NGS) have enabled the application of broad-scale sequencing in metagenomic studies [8]. This technique has been applied in various microbial niches including soil [9], human gut [10], marine [11].

In the current investigation we applied a suite of physical, chemical and metagenomic-based molecular approaches to analyze the accumulated matter and biofilm within a sand filter used in drinking water treatment. The study objectives were to (i) elucidate microbial community composition and function in the biofilm of sand filter and (ii) investigate the potential role of microbial community in the removal of aromatic compounds, inorganic N and heavy metals in drinking water.

## Materials and Methods

### Ethics Statement

No specific permits were required for the described field studies. The location is not privately-owned or protected in any way and the field studies did not involve endangered or protected species.

### 2.1 Sample Collection

Samples were obtained from a quartz-sand filter used at a drinking water plant, located in Henan Province, China. Underground water from the Yellow River Delta (80–120 m in depth) is the source water treated at this plant. The water quality was listed in Table S1 in File S1. The concentrations of NTU, Fe, Mn, and As exceed the drinking water limits [12]. The plant adopt a widely-used technological process for the groundwater treatment, i.e. drop aeration, sand filtration, and chlorination (Fig. S1 in File S1). After treatment, the water quality meet the drinking water standard of China (Table S1 in File S1).

The targeted sand filter is used for 10 y at the plant. Although there is a backwash per 48 h, the biofilm forming on the sand surface was obviously observed during the water treatment (Fig. S1 in File S1). We obtained eight sand mixtures (consisting of mature biofilm and associated particulates) from the filter system at 0, 10, 20, 30, 35, 45, 50 and 60 cm depths before backwash. All samples placed in sterile containers and stored with dry ice during transport to the laboratory in Beijing, China, where they were analyzed immediately.

### 2.2 Physicochemical Analysis

Physicochemical analysis of sand mixture samples was used to explore the available substrates and external environmental factors for microbial growth and metabolism.

Biofilm thickness was estimated using the radius of sand mixture subtracting the radius of sand. For chemical analysis, the sand mixtures were first dried with a freeze drier (LGJ-10B, China) prior to analysis. To determine dissolved organic carbon (DOC), 10 g dried sample were agitated with 20 ml deionized water for 20 h in a rotary shaker. The mixture was filtered through a 0.22 µm membrane filter and then analyzed with a TOC analyzer (Shimadzu TOC-V_CPH_, Japan). For ammonia-nitrogen (NH_3_-N) and nitrate-nitrogen (NO_3_^–^N) measurement, 5 g dried sample were mixed with 50 ml 1M KCl for 5 h in a rotary shaker. The mixture was filtered through a 0.45 µm membrane filter prior to analysis. The concentrations of NH_3_-N and NO_3_^–^N in the filtered extract were quantified using a standard spectrometric method [13] and ion chromatographic method (DIONEX ICS-2000, USA), respectively. Sample pH was determined with a pH meter (Thermo Orion 3 STAR, USA) in a sand mixture to water ratio of 1∶5 (w/w). Zeta potential was measured with a Nano particle sizing & Zeta potential analyzer (Delsa^TM^Nano, Beckman Coulter, USA). C, H, and N were analyzed with an element analyzer (Elementar Analysensysteme GmbH, Germany).

Total Fe, Mn, and As were extracted from the filter samples with standard procedure [13] and then measured using ICP-OES (Agilent 700, USA). Gas chromatograph-mass spectrometry (GC/MS), fourier transform infrared (FT-IR), and^ 13^C nuclear magnetic resonance (^13^C NMR) were used to identify the organic compounds present in the sand filter. X-ray photoelectron spectroscopy (XPS) was used to determine the distribution of elements. Full methods of GC/MS, FT-IR, ^13^C NMR, and XPS were described in Supplemental Materials and Methods in File S1.

### 2.3 DNA Extraction

DNA was extracted from 0.50 g fresh sand mixtures using the Power soil DNA isolation kit (Mobio, USA). Three replicate DNA extractions of individual sand mixture were stored at −80°C in a freezer for further molecular analysis.

### 2.4 Real-time PCR

Real-time polymerase chain reaction (PCR) was used to obtain the quantitative distribution of prokaryotes in the sand filter. Total bacteria, total archaea, nitrifier, and denitrifier were chosen as targets for this study.

Bacterial and archaeal 16S rRNA (representing bacteria and archaea), bacterial and archaeal *amoA* (representing ammonia-oxidizing bacteria (AOB) and ammonia-oxidizing archaea (AOA), respectively) [14], and bacterial *nosZ* genes (representing denitrifying bacteria) [15] were quantified by an ABI 7300 fast real-time PCR system based on the SYBR Green I method. The primer pairs and the thermal programs of the PCR amplification are described in the Table S2 in File S1. Plasmid standards and calibration curves construction were described in detail previously [15].

### 2.5 Metagenomic Analysis

Metagenomic analysis was used to characterize the composition and function of microbial community in the sand filter.

We merged 0–30 cm DNA samples and 35–60 cm DNA samples (at equal amount) into two individual metagenomic samples, labeled DZhigh (0–30 cm) and DZlow (35–60 cm), respectively. The same amount of DZhigh and DZlow metagenomic samples were used to construct two paired-end libraries (350 bp insert size) by using a Illumina TruSeq DNA Sample Preparation Kit (Illumina, Inc., San Diego, CA). Cluster generation was performed on an Illumina cBot automated cluster generation system according to the manufacturer’s instruction. The prepared templates were loaded on the Illumina Hiseq 2000 sequencing system for sequencing (90 bp pair-end reads). Sequence reads were initially filtered to remove adapters, low quality reads and reads that belonged to the host. The filtered reads (∼5 Gbp for both datasets) were assembled into long contigs using a velvet *de novo* assembler [16] with an optimum k-mer value on the cloud computing platform of the Beijing Computing Center (Beijing, China). The assembled contigs were uploaded to the MG-RAST (Meta Genome Rapid Annotation using Subsystem Technology, v3.2.2) website (http://metagenomics.anl.gov/) for taxonomic classification and function annotation using MG-RAST pipeline [17]. Microbial taxonomy was based on rRNA sequences determined using the M5RNA database, which can identify both prokaryotes and eukaryotes; the best-hit classification method was used. The minimum identity confidence value was set to 80% and the minimum alignment length was set to 50 bp. Taxonomic assignments at domain, phylum and class levels were analyzed for DZhigh and DZlow samples. For community function annotation, we focused on the metabolism of aromatic compounds, inorganic N, and heavy metals. For aromatic compounds and inorganic N, SEED subsystems and KEGG (Kyoto Encyclopedia of Genes and Genomes) annotation in MG-RAST with recommended parameters [18] (minimum alignment length, 50 bp; E-value cutoffs, 1e-5) were used to assign reads to different functional groups (SEED) or metabolic pathways (KEGG). Metabolic genes present in biofilm were identified and the abundances of those gene sequences were calculated. In addition, since most heavy metal transformation genes are not clearly annotated in the SEED and KEGG databases, we used Genebank as the reference database to search all the major genes involved in Fe, Mn and As transformation.

Statistical analysis of metagenomic profiles (STAMP) bioinformatics software [19] was used to compare community composition and function between the DZhigh and DZlow samples. Statistical significance was calculated using two-sided Fisher’s exact test, and the differences between proportions were analyzed using the Newcombe-Wilson method with 95% confidence interval.

Two original metagenomic datasets are archived at NCBI Sequence Read Archive (SRA) under the accession number of SRR747868.

### 2.6 Clone Library and Phylogenetic Analysis

Archaeal ammonia oxidation is thought to be very important to the removal of NH_3_ in water treatment from recent studies [14], [20], [21]. To verify whether the archaea in the sand filter were involved in ammonia oxidation, we performed clone library analysis to elucidate the composition of total archaea and AOA.

PCR amplifications for the archeal 16S rRNA and *amoA* genes were performed with the widely used primer pairs (Table S2 in File S1). Due to that PCR amplification of archaeal *amoA* from DZlow sample was weak and insufficient for constructing clone library, we only performed clone library analysis for DZhigh sample. PCR products were purified with the Qiaquick gel extraction kit and cloned into pGEM-T Easy vectors (Promega, USA). The recombinant plasmids were transformed into competent *E. coli* Top10. Twenty-five clones from 16S rRNA and thirty-five clones from *amoA* libraries were randomly selected and sequenced using an ABI 3730*xl* DNA Analyzer (Applied Biosystems, USA). Low -quality ends and vector contaminants were removed by the software SeqMan Pro software (DNASTAR). Ambiguous sequences were checked manually and excluded from further analysis. Operational taxonomic units (OTUs) were defined as groups where the sequence similarities were greater than 97%. OTU processing were performed using the Mothur software [22]. The OTU sequences were blasted against published gene sequences in the National Center for Biotechnology Information (NCBI) database. Phylogenetic trees were constructed using the neighbor-joining method with the software MEGA 5 [23].

All OTU sequences of archaeal 16S rRNA and *amoA* are deposited in the NCBI database under the accession numbers JX966094 (16S rRNA) and JX966095 to JX966099 (*amoA*), respectively.

## Results

### 3.1 Chemical Substance Distribution in the Sand Filter

The chemical matters retained in the sand filter were the potential available substrates for microbial growth and metabolism. Therefore it is essential to study the spatial distribution and characteristics of these chemical matters.

The physicochemical characteristics of the sand mixture sample at each site are shown in Table 1. Biofilm thickness, water content and the concentrations of C, N and H decreased gradually along the vertical sampling gradient. The potential harmful compounds including NH_3_−N, NO_3_^–^N and heavy metals (Fe, Mn, As) established the same decreasing trend. This revealed that the amount of chemical substances for microbial growth and metabolism had a gradually decreasing in vertical distribution in the sand filter. Zeta potential and pH did not demonstrate significant vertical differences.

**Table 1:** Physicochemical characteristics of the sand mixtures.

Samplesites	Estimated biofilmthickness (cm)	pH	Water content	C(mg/g)	N(mg/g)	H(mg/g)	DOC(mg/g)	Zeta (mV)	NH_3_−N(mg/g)	NO_3_^–^N(mg/g)	Fe(mg/g)	Mn(mg/g)	As(mg/g)
0 cm	0.075	7.80	77%	24.35	3.45	22.10	0.21	−13.43	0.00210	0.77	8.56	3.35	0.0012
10 cm	0.060	7.78	75%	18.10	3.15	16.60	0.20	−12.87	0.00196	0.75	6.35	3.28	0.0008
20 cm	0.050	7.80	74%	16.95	2.65	16.40	0.19	−12.75	0.00184	0.74	5.92	3.02	0.0005
30 cm	0.045	7.79	67%	13.95	2.30	14.30	0.19	−12.41	0.00121	0.67	4.83	2.83	0.0005
35 cm	0.035	7.81	53%	10.65	2.00	11.55	0.17	−11.65	0.00101	0.53	4.79	2.55	0.0003
45 cm	0.015	7.79	45%	11.60	1.80	11.75	0.13	−13.52	0.00087	0.45	4.16	2.08	0.0002
50 cm	0.050	7.81	21%	7.45	1.35	7.20	0.09	−12.29	0.00030	0.21	2.35	1.52	0.0001
60 cm	0.020	7.85	12%	3.05	1.25	3.30	0.09	−13.31	0.00007	0.12	2.32	1.35	0.0001

The numbers represent the mean values (2 or 3 replicates).

**Figure 1:**
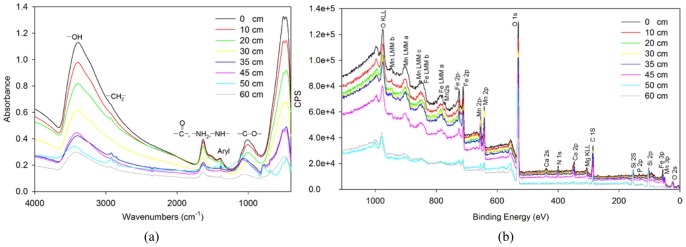
Chemical composition analysis of the sand filter based on (a) Fourier transform infrared (FT-IR) (b) X-ray photoelectron spectroscopy (XPS).

The composition of chemical substances in the sand filter was a key factor for affecting the composition and function of microbial community. So we used a suit of chemical methods to explore the organic and inorganic compositions of these chemical substances. For organic composition identification, GC/MS analysis with full-Scan model identified only a few long-chain alkanes such as hexadecane and dodecane in all samples as many overlap peaks existed in the GC/MS chromatogram; any aromatic compounds were not identified. The FT-IR spectra revealed the dominant organic functional groups in the sand mixture samples were OH–, CH_2_–, C = O (NH_2_– and –NH–), aryl, and –CO– as evidenced by peaks at around 3400 cm^−1^, 2920 cm^−1^, 1630 cm^−1^, 1400 cm^−1^, and 1010 cm^−1^, respectively (Fig. 1(a)). ^13^C NMR spectra (Fig. S2 in File S1) also revealed the presence of carbonyl C (C = O), aromatic C (aryl), alkyl C(CH_2_–) and O-alkyl C (–CO–) in the sand mixture samples. XPS analysis identified Fe, Mn, Ca and Mg in the sand mixture samples, in addition to O, C, N and P (Fig. 1(b)). These metals were also detected in the groundwater that supplies the plant (Table S1 in File S1). In summary, the results revealed that the organic (including aromatics) and inorganic compositions in the vertical profile were identical but their concentrations decreased proportionally along the vertical profile.

### 3.2 Microbial Biomass Distribution

**Figure 2:**
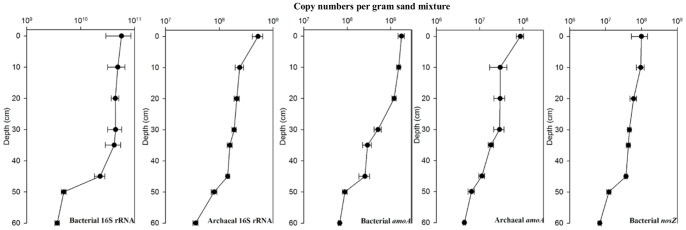
Vertical distribution of 16S rRNA (bacteria and archaea), *amoA*(bacteria and archaea), and *nosZ* gene in the sand filter system of the drinking water treatment facility. The results were obtained from real-time PCR assay. Error bars represent standard deviation from three independent experiments.

As with the physical and chemical parameters, a quantitative distribution of the bacteria and archaeal 16S rRNA genes (representing bacteria and archaea), bacterial and archaeal *amoA* (AOA and AOB (nitrifiers)) and *nosZ* (denitrifying bacteria) in the sand filter was observed (Fig. 2). The density of prokaryotic material decreased gradually with an increase in depth. Bacteria were dominant in the sand filter. The density of the bacterial 16S rRNA gene was 10^9^–10^11^ copies per gram of sand filter, which was 61–267 times the measured quantity of archaea. AOB abundance was significantly higher than AOA (AOB:AOA ratio of 14 to 51), suggesting that AOB may be primarily responsible for ammonia oxidation. The *nosZ/*bacterial 16S rRNA ratio ranged from 0.1% to 0.3% in the sand filter.

### 3.3 Microbial Community Composition and Function

Metagenomic analysis was used to elucidate the microbial community composition and function in the biofilm of the sand filter. After velvet assembly, 689,291 sequences in the DZhigh library, with an average length of 311 bps, and 759,508 sequences in the DZlow library with an average length of 315 bps, were obtained. These two assembled metagenomic datasets were uploaded to MG-RAST for taxonomic classification and function annotation. A total of 468 rRNA sequences (DZhigh, 231; DZlow, 237) retrieved from datasets were used to identify the taxonomic affiliation; 1,398,747 protein-encoding sequences (DZhigh, 666,253; DZlow, 732,494) were used to identify community function.

#### 3.3.1 Community composition

**Figure 3:**
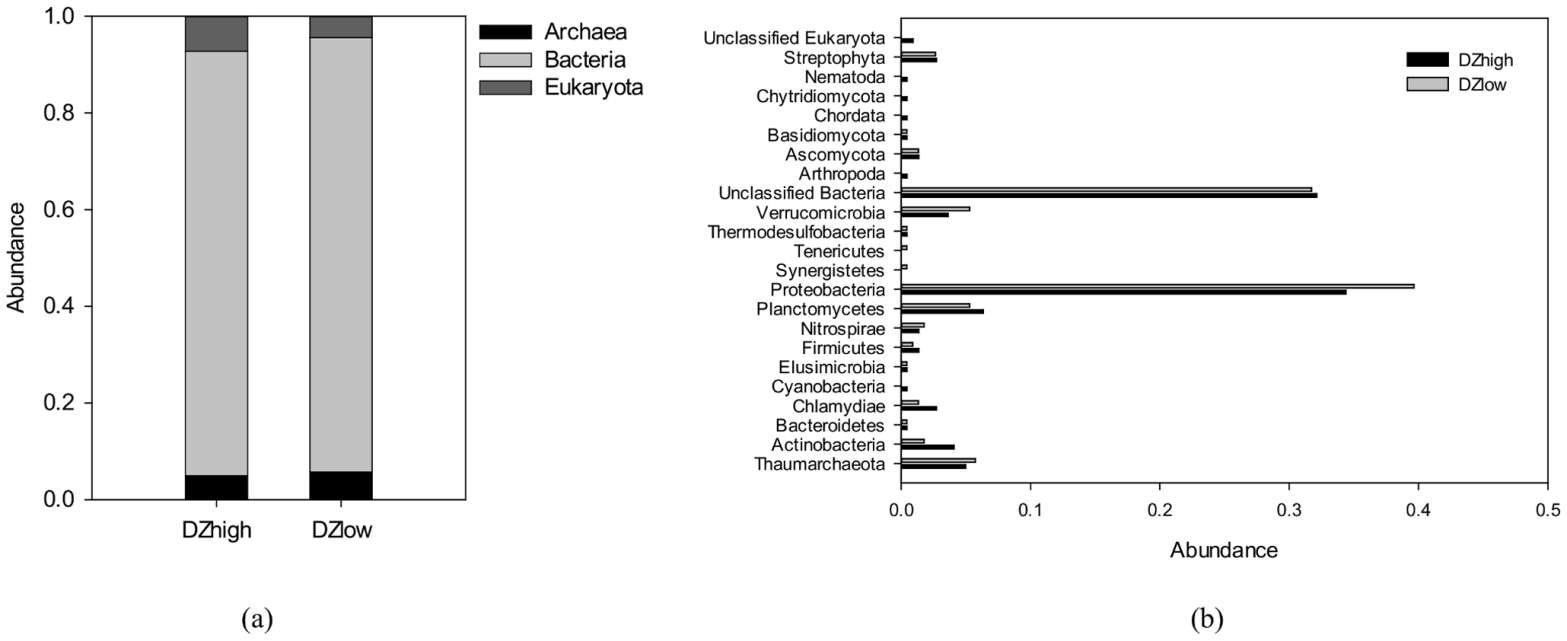
Taxonomic classifications of microbial communities in the sand filter biofilm at (a) domain level and (b) phylum level. The data was obtained based on metagenomic data revealed by Illumina.

Taxonomic classification of rRNA sequences were performed for assessing microbial community membership (Fig. 3). Bacteria were dominant among three domains, which accounted for 87.8% of DZhigh and 89.9% of DZlow (Fig. 3(a)). Archaea and eukaryotic organisms ranged from 4% to 7% in the filter biofilm. As shown in Fig. 3(b), Thaumarchaeota was the sole phylum in the archaea domain. The bacterial community, however, was more diverse, with 13 different phyla across two samples (11 in DZhigh, 12 in DZlow). Proteobacteria (34.4% in DZhigh, 39.6% in DZlow) was the most abundant bacterial phylum in both samples. Seven positively-identified eukaryotic phyla (seven in DZhigh, three in DZlow) were found. Fungi such as Ascomycota and Basidiomycota were present in both samples (2.3% in DZhigh and 1.8% in DZlow). Streptophyta, which are land plants, were also present in both samples, with 2.7% in DZhigh and 2.6% in DZlow. Some Arthropoda, Chordata, and Nematoda were observed in the DZhigh sample, having been captured during the filtering process (Fig. 3b).

The Proteobacteria in the two samples consisted of six classes (Alpha-, Beta-, Gamma-, Zeta-, Epsilon-, and Delta-Proteobacteria) plus unclassified Proteobacteria (Fig. S3 in File S1). Alphaproteobacteria were dominant in both samples.

Statistical analysis conducted at the class level found no significant differences between DZhigh and DZlow (Fig. S3 in File S1).

#### 3.3.2 Community function annotation

Function annotation provided a “snapshot” assessment of the genetic potential of the microbial communities residing in the sand filter biofilm. Using hierarchical analysis of SEED Subsystems in MG-RAST (Fig. S4 in File S1), 1.3% (DZhigh) and 1.4% (DZlow) of annotated sequences were associated with metabolism of aromatic compounds; 1.0% (for both DZhigh and DZlow) were associated with nitrogen metabolism. Using KEGG mapper (Fig. S5 in File S1), we found two datasets (DZhigh and DZlow) that encompassed most of the metabolic pathways annotated with KEGG; there was a slightly different annotation coverage between two datasets.

In this metagenomic study we focused on metabolism genes and pathways associated with aromatic compounds, inorganic N and heavy metals due to the potential human health toxicity associated with these constituents.

##### (i) Aromatic compounds degradation

The community functional genes involved in aromatic compound metabolism from SEED and KEGG databases are shown in Table 2. Twenty six (26) (DZhigh, 16; DZlow, 15) types of aromatic compound degradation pathways were identified. Chloroaromatic, polycyclic aromatic, benzoate and toluene degradation genes were present in both datasets. Most of the identified aromatic degradation genes in both databases were those of easily biodegradable compounds, e.g. caprolactam, toluene, benzoate and phenylalkanoic. Persistent or emerging aromatic contaminants, such as polycyclic aromatic, heterocyclic aromatic and pharmaceutical antibiotics had comparatively lower occurrence of degradation genes in the sand filter. The common aromatic metabolism enzymes, aromatic oxygenase (monooxygenase and dioxygenase) and dehydrogenase, demonstrated a higher rate of abundance among the annotated aromatic enzymes. The inventory of aromatic oxygenases and dehydrogenases involved in activation and cleavage of the aromatic ring was extensive and phylogenetically diverse in the sand filter.

**Table 2:** Relative abundance of aromatic compound metabolism-related gene sequences retrieved in two metagenomic datasets.

SEED			KEGG		
Compounds	DZhigh	DZlow	Compounds	DZhigh	DZlow
n-Phenylalkanoic acid	392	436	Caprolactam	115	148
Benzoate	209	224	Toluene	109	118
Phenylacetyl-CoA	40	58	Benzoate	106	103
Gentisare	26	23	Nitrotoluene	69	135
Phenylpropanoid	21	19	Naphthalene	66	62
Chloroaromatic	12	0	Polycyclic aromatic hydrocarbon	57	19
Quinate	12	14	Styrene	47	36
p-Hydroxybenzoate	8	6	Aminobenzoate	40	44
Aromatic Amin	5	8	Bisphenol	22	24
Biphenyl	1	0.3	Ethylbenzene	16	14
Chlorobenzoate	1	1	Atrazine	6	9
Toluene	1	0	Chlorobenzene and chlorocyclohexane	6	0.1
Cresol	0.6	0.3	Xylene	2	2
Salicylate ester	0.4	0.3	Dioxin	0.3	0
Carbazol	0	0.3	DDT	0.1	0
p-cymene	0	0.3			

Values are normalized by randomly sampling 100,000 assembled sequences per sample.

##### (ii) Inorganic nitrogen removal

Gene sequences associated with nitrogen metabolism, especially nitrification and denitrification processes, were included in this assessment because of their critical roles in nitrogen removal. The complete nitrification (*amo*, and *hao*) and dentrification (*nar*, *nir*, *nor*, *nos*) genes were found in two metagenomic datasets (Table 3). Ammonia oxidation (*amo*) is the first and rate-limited step in nitrification, and was considered to be catalyzed by AOB and AOA [24], [25]. From the analysis of *amo* gene sequences in two metagenomic datasets, we found the AOB community in the sand filter consisted primarily of two genera, *Nitrosospira* (30.5% from SEED; 58.10% from KEGG) and *Nitrosomonas* (66.5% from SEED; 41.9% from KEGG). only one sequence (<1% annotated *amo* sequences) were affiliated with AOA, which indicated that AOB played a dominant role in ammonia oxidation. For denitrification, the annotated *nar* and *nir* gene sequences were more abundant in both datasets, most likely due to a high NO_3_^−^ concentration in the sand filter. The annotated denitrification sequences were affiliated with diverse bacteria phyla including Bacterodetes, Verrucomicrobia and Proteobacteria, the latter being dominant.

**Table 3:** Relative abundance of nitrification- and denitrification-related gene sequences retrieved from two metagenomic datasets.

Nitrification/denitrification	Transformation enzyme	SEED		KEGG	
		DZhigh	DZlow	DZhigh	DZlow
Nitrification	Ammonia monooxygenase (*amo*)	15	12	11	9
Nitrification	Hydroxylamine oxidoreductase (*hao*)	13	10	18	18
Denitrification	Nitrate reductase (*nar*)	83	95	23	22
Denitrification	Nitrite reductase (*nir*)	51	27	41	34
Denitrification	Nitric-oxide reductase (*nor*)	3	11	23	15
Denitrification	Nitrous-oxide reductase (*nos*)	15	19	11	10

Values are normalized by randomly sampling 100,000 assembled sequences per sample.

##### (iii) Heavy metals transformation

Fe, Mn and As were the heavy metals of choice for assessment due to that they are widely present in groundwater, and are harmful to human health in excess amounts. The results of MG-RAST BLAT with Genebank identified sequences associated with ferric reductase (six hits from DZhigh, one from DZlow), suggesting that ferric iron reduction was occurring in the sand filter. There was no evidence of ferrous oxidase due to pre-sand filtration aeration which converts ferrous iron to ferric iron. Although data exist that suggested the likelihood of microbial oxidation of Mn^2+^ (dissolved Mn^2+^ to insoluble Mn^4+^) in the sand filter [26], we did not find any sequences directly associated with Mn oxidation, although some sequences were annotated with manganese transport genes. The abundance of arsenite oxidase genes (6.24 genes per 100,000 assembled sequences from DZhigh; 2.90 from DZlow) were higher than arsenate reductase genes (0.58 from DZhigh; 0.79 from DZlow), revealing arsenite oxidation might be the dominant reaction in arsenic biological transformation. These arsenite oxidase-encoding genes were found to be affiliated with Proteobacteria, Nitrospirae, Chlorbi, Chloroflexi and Crenarchaeota (Archaea).

### 3.4 Clone Library and Phylogenetic Analysis for Archaea and AOA

**Figure 4:**
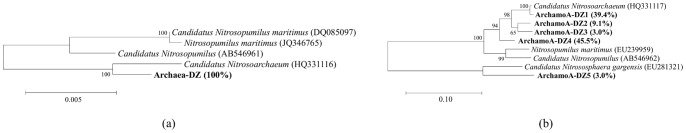
Phylogenetic trees based on the (a) archaeal 16S rRNA and (b) archaeal *amoA* representative sequences (OTUs, 97% similarity) from the DZhigh sample. The numbers on the branch nodes represent percentage of bootstrap resamplings based on 1000 replicates (only ≥50% are shown). The scale bar indicates the number of nucleotide substitutions per site. The relative abundance of each OTU in each clone library is shown in parentheses.

After metagenomic analysis, we could not conclude that archaea were involved in the ammonia oxidation as the archaeal *amo* sequences were quite scarce (only one sequence) in metagenomic datasets. To further verify this conjecture, we also performed clone library analysis based on archaeal 16S rRNA and *amoA* genes (Fig. 4) to elucidate the compositions of archaea and AOA. Good’s coverage were 100% (archaea) and 93.9% (*amoA*), which indicated that these two clone libraries covered the most diversity of community. As shown in Fig.4(a), 22 archaeal 16S rRNA sequences were identified to only one OTU which was affiliated with *Nitrosoarchaeum* (99% similarity) belonged to Thaumarchaeota. Thirty-three archaeal *amoA* sequences were identified to 5 OTUs, and were similar to those *amoA* sequences retrieved from *Nitrosoarchaeum* or *Nitrososphaera* (belonged to Thaumarchaeota )(Fig. 4(b)). The above results demonstrated that the the archaea in the sand filter were likely involved in ammonia oxidation.

## Discussion

Few published studies have focused on the role of microbes in drinking water treatment using sand filters, probably due to frequent backwashes and low substrate levels that are considered antagonistic to substantial colonization. However, microbial activity on the sand surface may be quite significant as biofilm develops under moderate temperature conditions [27]. Our study demonstrated the biofilm formed within 2 days was comparatively thick (up to 0.75 mm, nearly the same as the media) in the sand filter and the microbial densities (reaching ∼10^11^ prokaryotes per gram) were also high (Fig. 2). The interstitial microbial biomass decreased with sampling depth, corresponding to a decrease of organic or inorganic compounds concentrations as these compounds may act as a nutrient source for many microbial taxa; this is consistent with previous study [2]. However, pH and the composition of compounds did not change demonstrably with depth (Table 1 and Fig. 1), resulting in a high degree of similarity in the microbial communities at different depths (Fig. S3 in File S1).

For taxonomic classification of the microbial community, both rRNA and protein-coding sequences from metagenomic datasets indicated Alphaproteobacteria was the dominant group in the filter biofilm. Alphaproteobacteria includes numerous phototrophs, chemolithotrophs, chemoorganotrophs and aerobic photoheterotrophs [28], and are widely distributed in the aerobic [29], [30] and anaerobic biofilm [31] found at drinking water or wastewater treatment plants. Thaumarchaeota was found to be the only archaeal phylum in the biofilm from the metagenomic and clone library analysis. This phylum is among the most abundant archaea on Earth and is well known for its ability to oxidize ammonia [32], [33]. Our results also demonstrated that the Thaumarchaeota in the sand filter were likely involved in ammonia oxidation (Fig. 4).

In our study, FT-IR spectra and ^13^C NMR analysis demonstrated the existence of aromatic compounds in the sand filter and their concentrations decreased after the treatment. Furthermore, the metabolic profiles from metagenomic datasets revealed a broad array of key enzymes or genes related to the utilization and mineralization of aromatic compounds (Table 2). These results indicated the microbes in the sand filter biofilm played an important role in the removal of aromatic compounds in drinking water. In addition, a challenge for the environmental researcher is to obtain detailed information on aromatic compounds and their metabolic pathways in a drinking water treatment system. It may be difficult to derive such information by chemical analyses alone due to the low concentrations and complexity of target constituents. In recent years more and more evidence proved that there is a strong relationship between the aromatic degradation gene frequency and the aromatic degradation rates [9], [34], [35]. The aromatic degradation genes can be regarded as an indicator to predict the possible aromatics and their potential metabolic pathways. By relying on those degrading-enzymes or genes with high abundances, we may predict the possible aromatic compounds in the sand filter (even in groundwater) and their potential metabolic pathways. For example, benzoate whose degradation genes present in the sand filter with high abundance (Table 2), is a central intermediary compound in the anaerobic and aerobic metabolism of various aromatic compounds, such as toluene, xylene, carbazole and biphenyl [36]. According to that, the metabolic pathway “from xylene to benzoate” is highly suspected to be occurring in the sand filter as the related transformation genes were abundant in the system. Therefore, metagenimic method can be regarded as an alternative tool to predict the potential aromatic metabolic pathways in the drinking water treatment.

Groundwater often contains high concentrations of ammonia, iron and manganese, making it unsuitable for direct use as drinking water [37]. Our results from N metabolism gene analysis (Table 3 and Fig. 2) indicate that ammonia-N removal in the groundwater likely occurs through nitrification, followed by denitrification. The nitrification and denitrification processes were achieved simultaneously in our sand filter and thus were of great benefit for total nitrogen removal [38]. The competitive role of AOA and AOB in ammonia oxidation has been the focus of considerable research in recent years. In our study, the abundance of AOB was significantly higher than AOA (Fig. 2), indicating AOB might be dominant in ammonia oxidation and thus be mainly responsible for the NH_3_ removal.

Iron removal in the drinking water was always achieved with physicochemical methods, e.g. aeration. But for manganese removal, naturally-occurring manganese-oxidizing bacteria colonize sand filters through which Mn^2+^-containing water was passed, resulting in efficient oxidation of soluble Mn^2+^ [39]. The oxidation products, i.e. insoluble tetravalent manganese oxides, were then captured in the sand filter (Fig. 1(b)). However, we did not identify any genes related to manganese oxidation by searching against databases of known genes. This failure is likely because manganese oxidation genes in public databases are incomplete and there is considerable uncertainty associated with several proposed manganese oxidation genes [40], [41]. Arsenic contamination occurs in groundwater sources in many countries, including China [42]. In our study, the comparatively higher abundance of arsenite oxidase indicates that most arsenite in the groundwater was likely converted to the more strongly sorbing, generally less mobile and less toxic arsenate species in the sand filter [43]. This transformation guaranteed the arsenic in effluent from the sand filter could meet the drinking water quality standard.

In summary, using integrated metagenomic and physicochemical analyses, our study demonstrated that microbial activities play a crucial role in harmful material removal of drinking water in the sand filter. This knowledge can help us to formulate a study plan focused on constructing suitable microbial communities in sand filters for drinking water treatment and harnessing their unique water purification characteristics.

## Supporting Information

File S1 **Combined file of all supporting information.** Supplemental Materials and Methods. Figure S1 Schematic diagram of Dongzhou drinking water treatment. A portion of sand mixtures in eight different depth zones (0, 10, 20, 35, 45, 50, 60 cm) was taken out from the quartz-sand filter unit (grey) for our study use. The photo shows the difference of dimension between 0 cm and 60 cm sand mixtures. Figure S2^ 13^C NMR analysis of the sand mixture (0 cm sample). Figure S3 Composition analysis of microbial community at class level in MG-RAST server [8] using a maximum e-value of 1e-5, a minimum identity of 80%, and a minimum alignment length of 50. Results were plotted and analyzed using STAMP software [9]. Blue: DZhigh; Yellow: DZlow. Statistical significance was calculated using two-sided Fisher’s exact test, and the differences between proportions were analyzed using the Newcombe-Wilson method with 95% confidence interval. Figure S4 Hierarchical analysis of metagenomic data annotated to SEED *subsystems* in MG-RAST server [8] using a maximum e-value of 1e-5, a minimum identity of 60%, and a minimum alignment length of 50. The results were plotted and analyzed with STAMP software [9]. Blue: DZhigh; Yellow: DZlow. Statistical significance was calculated using two-sided Fisher’s exact test, and the differences between proportions were analyzed using the Newcombe-Wilson method with 95% confidence interval. Figure S5 KEGG metabolic pathways of two metagenomic samples. Purple: identical pathway of DZhigh and DZlow; Blue: unique pathway of DZhigh; Red: unique pathway of DZlow. Table S1 Water quality of influent (groundwater) and effluent (after sand filter treatment) of Dongzhou drinking water plant in sampling month (numbers represent the mean values). Table S2 PCR primer pairs and thermal programs used in this study.
(RAR)
